# Award of the Gross Prize of One Thousand Dollars

**Published:** 1902-03

**Authors:** 


### AWARD OF THE GROSS PRIZE OF ONE THOUSAND DOLLARS.

"The Philadelphia Academy of Surgery, as trustees of the Samuel D. Gross Prize for original research in surgery, of one thousand dollars, have awarded this prize, after six years interval, to Dr. Robert H. M. Dawbarn, of New York City.

"The treatise which won the competition was entitled 'The Treatment of Certain Malignant Growths by Excision of both External Carotids.' Upon this topic Dr. Dawbarn has worked, as opportunity served, for seven years past. The essay when published will contain the histories, with pathologists' report in each instance confirming diagnosis of malignancy, and specifying its variety, of forty carotid extirpations by the author himself, and as many additional by about a dozen other surgeons. At least two of these are members of the Philadelphia Academy of Surgery.

"By the terms of Dr. Gross's bequest the prize essay must be published in book form and a copy thereof be deposited in the Samuel D. Gross Library of the Philadelphia Academy of Surgery."—*Journal of the American Medical Association.*

It is with unusual pleasure that the foregoing is given a prominent place in this journal. Dr. Dawbarn is an active member of The New York Institute of Stomatology, and has contributed largely to its very pronounced success as a dental organization. He is one of the few great surgeons who feel that much may be gained by a closer intimacy with dentists and their work. This broad conception is so unusual that it is worthy of special record.

The writer has long been familiar with Dr. Dawbarn's extended surgical experience, and it is not, therefore, surprising that he succeeded in securing the prize for original research in surgery. The congratulations of this journal are extended to him for his labor and the result.

